# Supplementary figures and images for: Lack of the MHC class II chaperone H2-O causes susceptibility to autoimmune diseases
Source: PLoS Biol. 2020 Feb 18;18(2):e3000590. doi: 10.1371/journal.pbio.3000590 (PMC7028248; doi:10.1371/journal.pbio.3000590)

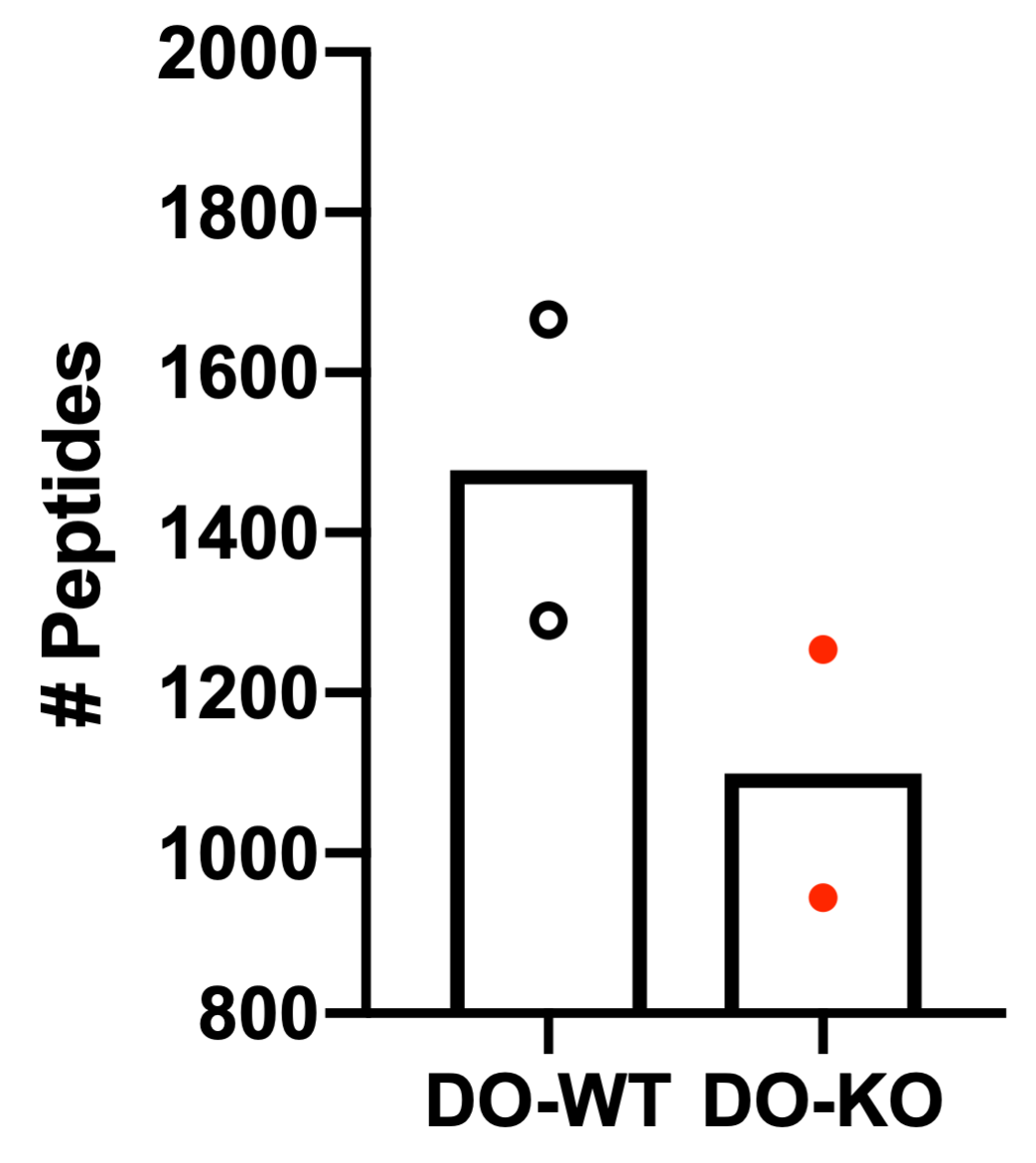

Supplement: S1 Fig — Lower numbers correspond to the first experiment utilizing 5 mice per group (944 KO versus 1,254 WT). Upper numbers correspond to a replicate experiment using 10 mice per group (1,290 KO versus 1,666 WT). Experimental results depicted in this figure can be found in S1 Data. KO, knockout; WT, wild-type. (TIF) [file pbio.3000590.s001.tif]

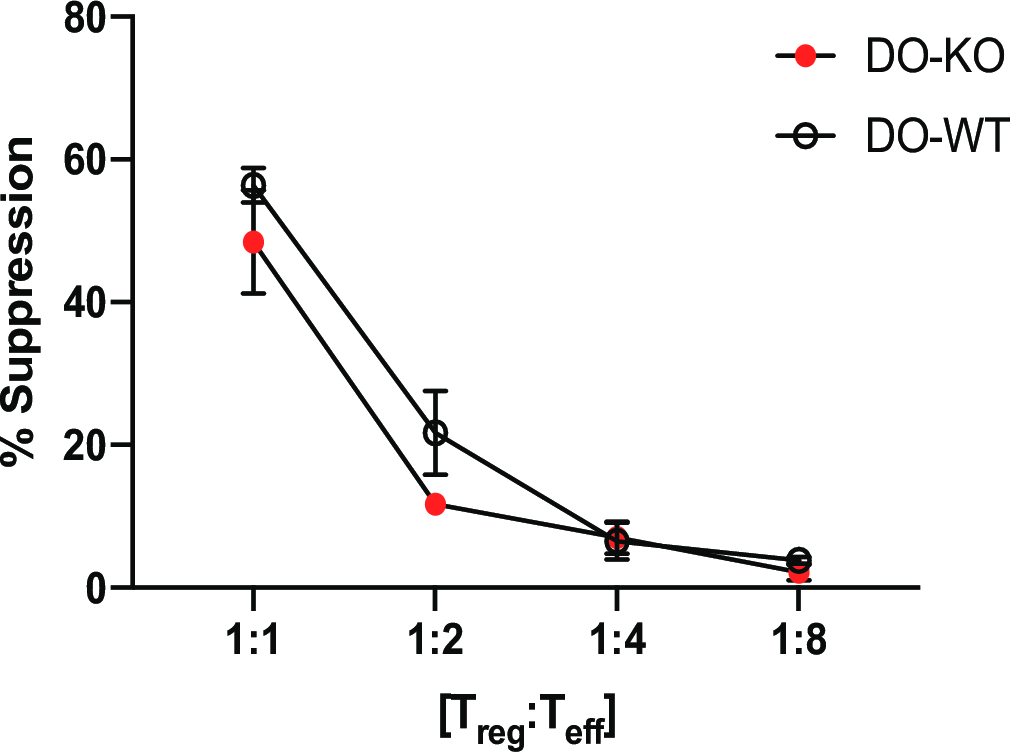

Supplement: S2 Fig — Tregs from naïve DO-WT (white) and DO-KO (red) mice were plated at varying concentrations (1:1, 1:2, 1:4, 1:8) with proliferation dye–labeled naïve CD4 T cells in the presence of irradiated APCs with soluble CD3 for 72 hours. The amount of suppression was calculated as follows: %Suppression=(%ofproliferatedresponderswithnoTreg−%ofproliferatedresponders)%ofproliferatedresponderswithnoTreg*100. Representative curve of 3 individual replicate suppression assays. Experimental results depicted in this figure can be found in S9 Data. APC, antigen-presenting cell; DO, H2-O; KO, knockout; Treg, regulatory T cell; WT, wild-type. (TIF) [file pbio.3000590.s002.tif]

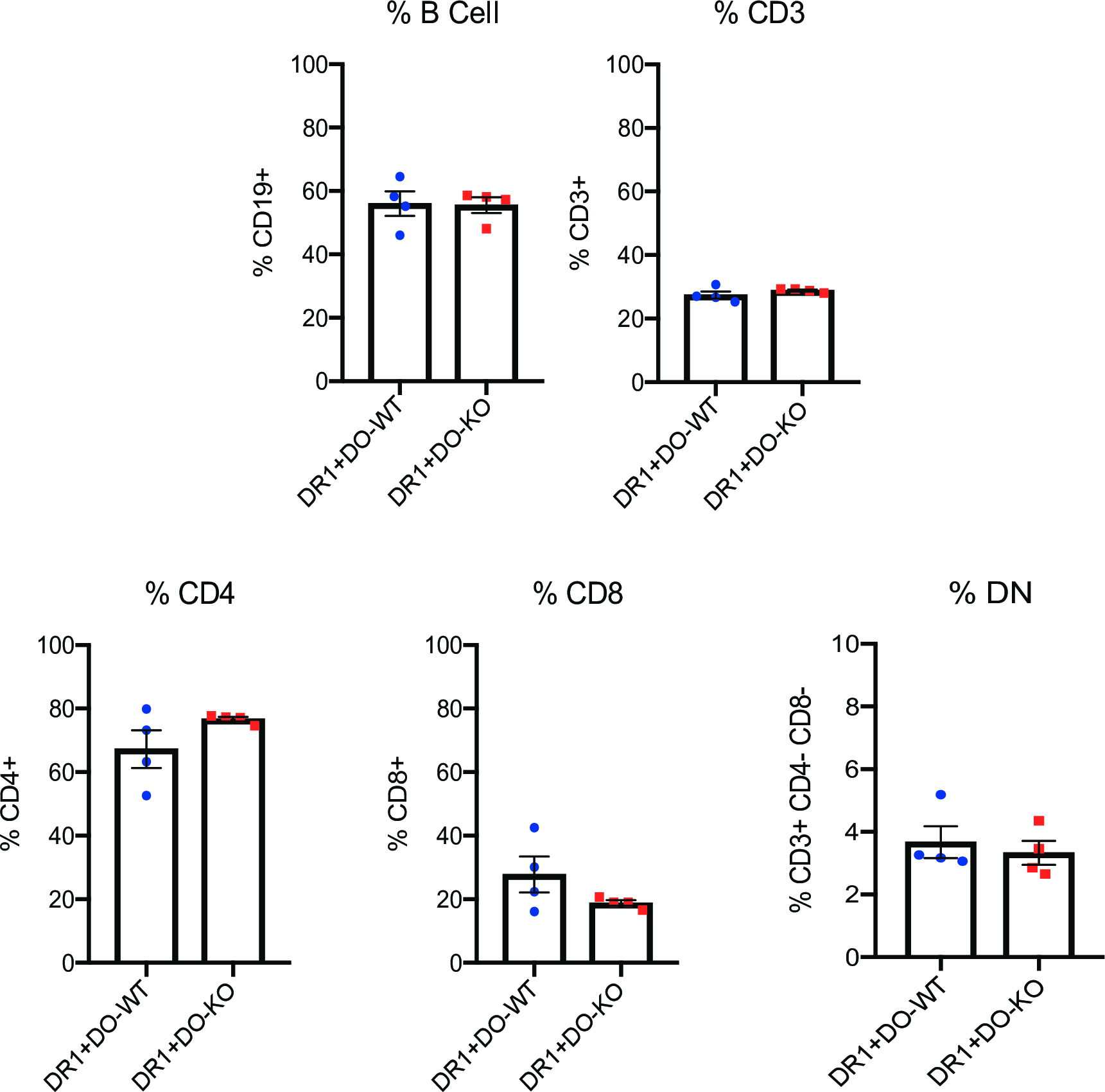

Supplement: S3 Fig — Similar cellular distribution in lymphoid tissues in naïve DR1+DO-WT (Blue) and DR1+DO-KO (Red) mice. Compiled graphs showing characterization data from 4 individual DR1+DO-WT and 4 DR1+DO-KO mice. Experimental results depicted in this figure can be found in S10 Data. DO, H2-O; DR1, HLA-DR1; KO, knockout; WT, wild-type. (TIF) [file pbio.3000590.s003.tif]

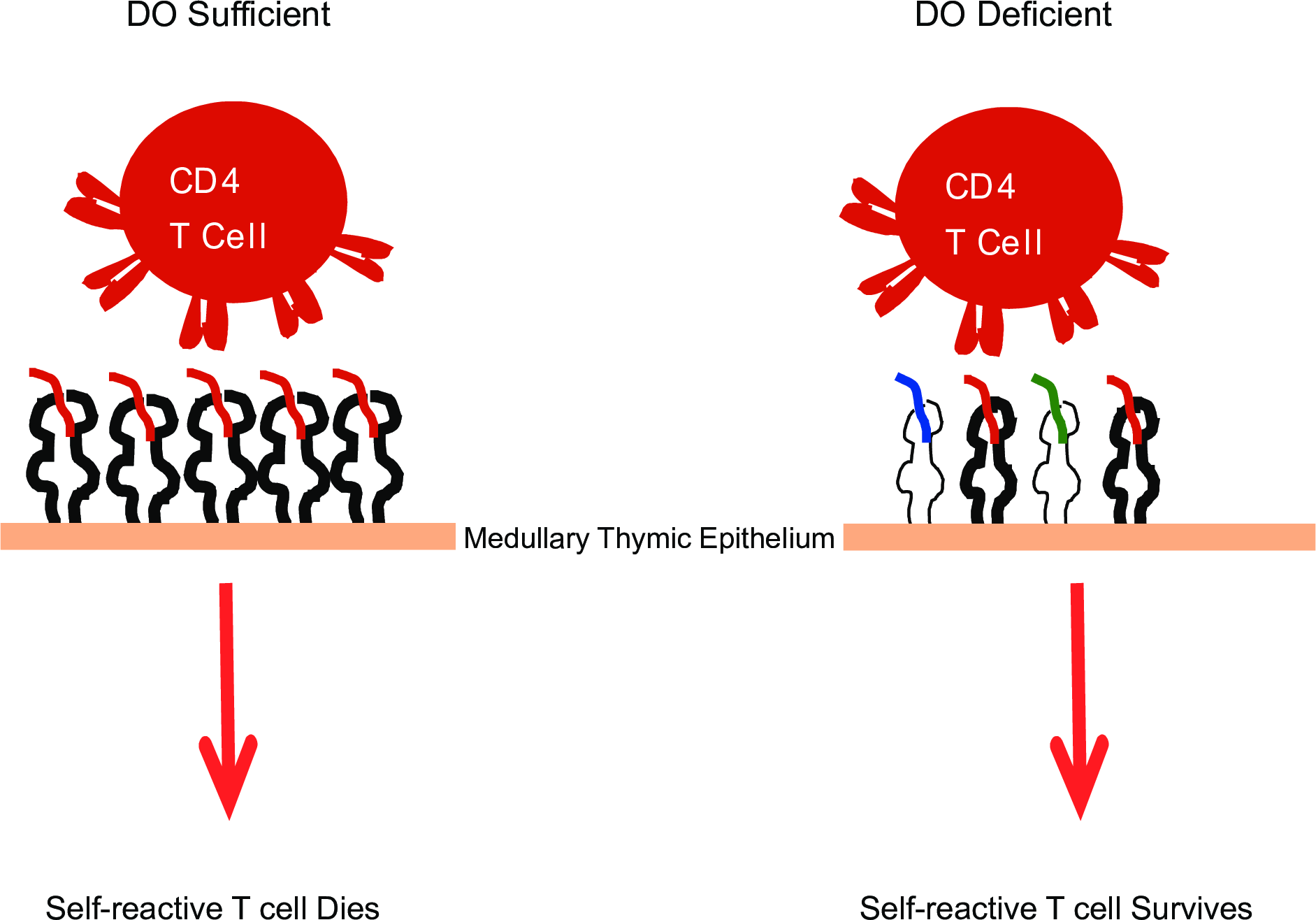

Supplement: S4 Fig — Illustration of proposed model of how negative selection would be impacted in the presence of DO (left) or in its absence (right). A higher density of the cognate epitope leads to successful negative selection and, conversely, absence of DO would cause a faulty negative selection. DO, H2-O. (TIF) [file pbio.3000590.s004.tif]

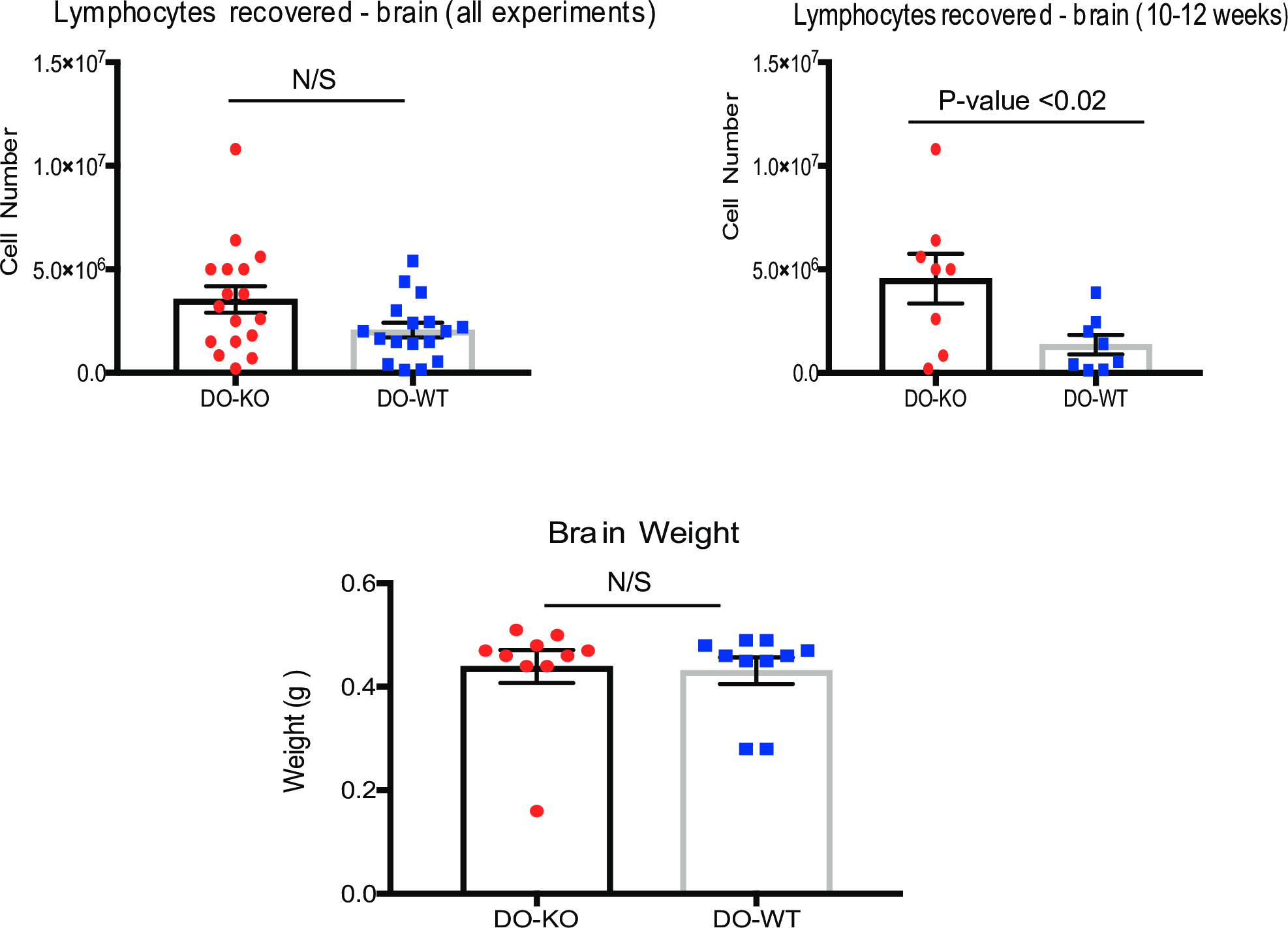

Supplement: S5 Fig — Total number of lymphocytes recovered from brains of diseased DO-KO or DO-WT mice. Diseased mice from all experiments (top left) or diseased mice from the 10–12-week time point (peak differences in disease scores, top right). Diseased brains of DO-KO and DO-WT mice weighed the same (bottom left). Each dot represents an individual mouse. Data represented as mean ± SEM. Experimental results depicted in this figure can be found in S11 Data. DO, H2-O; EAE, experimental autoimmune encephalomyelitis; KO, knockout; WT, wild-type. (TIF) [file pbio.3000590.s005.tif]

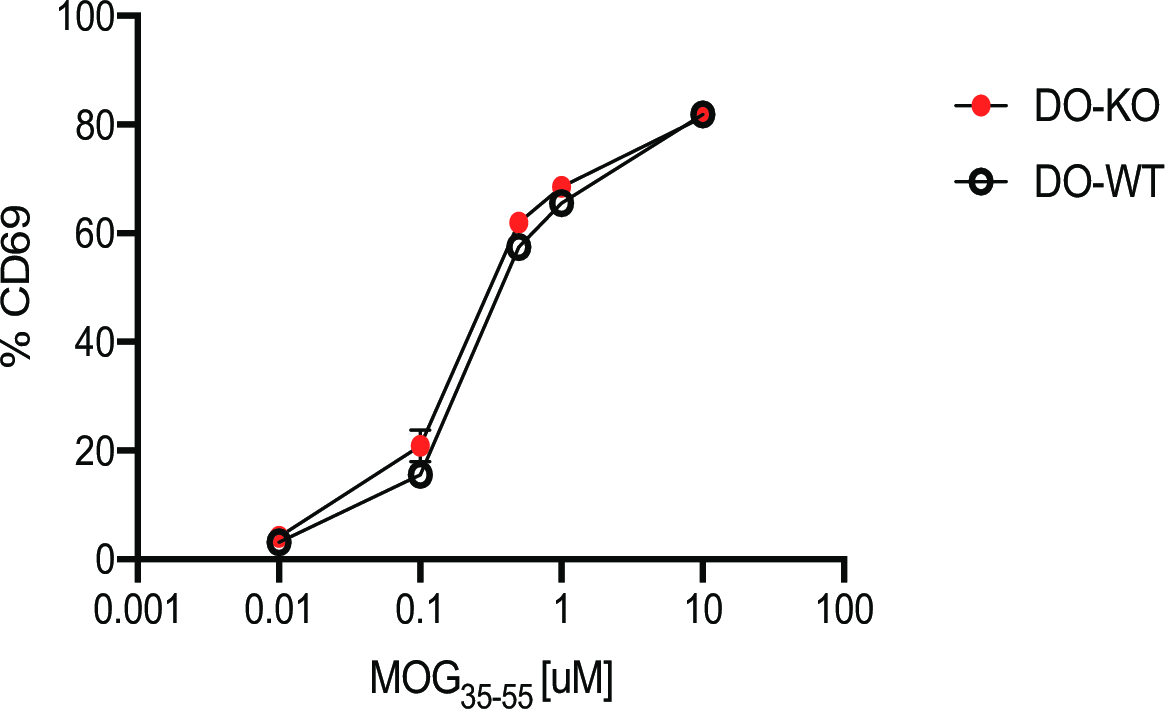

Supplement: S6 Fig — Isolated B cells from DO-KO (Red) and DO-WT (White) were pulsed with various concentrations of MOG35–55 peptide and cultured with isolated 2D2 CD4 T cells for 48 hours. Cells were then assessed for activation by up-regulation of CD69 in both groups. As shown, 2D2 T cells cocultured with B cells from either strain led to an almost linear increase in CD69 expression, indicating no differences in the level of I-A(b) between the two strains. Experimental results depicted in this figure can be found in S12 Data. DO, H2-O; KO, knockout; MOG, myelin oligodendrocyte glycoprotein; WT, wild-type. (TIF) [file pbio.3000590.s006.tif]
