# Supplementary material for: Lack of the MHC class II chaperone H2-O causes susceptibility to autoimmune diseases
Source: PLoS Biol. 2020 Feb 18;18(2):e3000590. doi: 10.1371/journal.pbio.3000590 (PMC7028248; doi:10.1371/journal.pbio.3000590)
Supplement: Attachment — Submitted filename: Point by point Response.docx [file pbio.3000590.s022.docx]

**Point-By-Point Response to the Reviewer Remarks**:

**Reviewer #1:**

Comments in detail:

•	*Although it is correct that in vivo studies of mice over-expressing or lacking H2O partly reveal complex effects (not surprisingly given that editing will modulate presentation of individual peptides in complex ways), the literature contains a fair amount of data strongly suggesting that H2O acts as competitor for H2M, thus inhibiting editing by H2M. A key structural paper in this context, published by Guce and Stern, is not cited.*

**Response: We apologize for the omission; Guce et al paper has been addressed in the revised version.**

•	*The notion that H2O modifies susceptibility to autoimmune disease has already been introduced by the demonstration that NOD mice overexpressing H2O are protected from type 1 diabetes, which would be expected to be mentioned.*

**Response: We have mentioned this paper in the revised version.**

•	*Peptide elution experiments are known to produce poorly reproducible results, so that multiple rather than a single experiment would be important to confirm the data in Fig. 1.*

**Response: Our presented data are representative of two completely independent experiments, which was mentioned in the legend of that Figure.**

- *Affinity data should be presented as mean IEDB scores rather than better or worse than CLIP.*

**Response: We changed the presentation of the data to the absolute *IEDB* score to find an even better correlation with predicted lower affinities for DO-KO eluted peptides.**

- *While it is technically difficult or impossible to elute peptides from TECs, the statement that use of splenic B cells in their place is justified by “similarity in antigen processing” would need to be supported by evidence or an appropriate citation.*

**Response: We apologize for the omission, and in the revised manuscript several published papers that document antigen processing compartments in mTEC include DM, MHC II, MIIC and processing cathepsins are included.**

•	*Fig. 2 shows that WT CD4 T cells mount a stronger proliferative response to KO splenocytes than the reverse. Given that WT splenocytes presented, according to Fig. 1, more unique peptides, they are expected to be more antigenic for KO CD4+ T cells rather than the reverse. Thus, this result is unexpected and should at least be discussed.*

**Response: We think that in the presence of DO, immunodominant peptides are presented at higher abundance, hence are better detected. Whereas, steps in antigen preparation and detection by MS might lead to reduction/elimination of eluted peptides present in lower abundance, a scenario expected to be at play for our DO-KO samples. A seemingly lack of correlation between the higher numbers of unique peptides eluted in Fig 1 and a lower proliferation of DO-KO T cells against DO-WT stimulation may be reflective of the higher sensitivity of T cells for detecting antigens. We have made sure that this point is clearly addressed in the revised discussion section.**

•	*While the experiment in Fig. 2 indeed shows that, not surprisingly, WT and KO present different peptide repertoires, this does not indicate that H2O-KO CD4+ repertoire includes autoreactive cells (it is also unclear why the same is not proposed for the reverse response). The unique peptides absent from KO splenocytes are more appropriately considered “allo-type” rather than “self” peptides. Thus, no conclusion regarding autoreactivity can be deduced from this experiment.*

**Response: We disagree. We wish to clarify that the statement that H2O-KO mice harbor increased levels of autoreactive CD4 T cells is based upon the right panel of Figure 2b (currently 3C). This experimental condition was simply KO mice responding to KO cells. This is not an “allo-type” response since our DO-KO mice have been bred to homozygosity. If this was truly an “allo-type” response, then we would expect to see similar proliferation from the WT mice immunized with WT cells (current Figure 3C, right panel), which is clearly not true.**

•	*The authors determined TCR repertoires after 4 months of continued restimulation without justifying this very long delay. Extensive culture and restimulation are likely to produce a TCR repertoire deviating largely from the in vivo repertoire, reducing the information that can be derived from these experiments.*

**Response: Continuing the culture of the stimulated CD4 T cells for 4 months had at least two purposes:**

a) **First, we wanted to enrich for only those cells that proliferated to their specific ligand presented by B cells from the other strain. This, we hoped, would allow generating cell lines specific for the unique peptides identified by peptide elution. However, using the T cells to identifying their specific peptide proved too difficult.**
b) **Second, having an enriched T cell pool before sequencing, we felt, would improve our ability to detect the differences caused by loss of DO function.**

**Nonetheless, we removed the 4-months culture data from the current version of the manuscript.**

•	*The authors interpret the somewhat higher number of unique TCRs found in H2O ko CD4+ T cells as evidence of lack of thymic deletion. However, since WT splenocytes express a much higher number of unique and therefore antigenic peptides, T cells from H2O ko mice are likely to engage a broader T cell repertoire in their response. Thus, at least two and probably additional explanations for this difference are possible. Moreover, no statistical evaluation is provided to show that this difference is significant. The additional interpretation of Fig. 2, proposing that TCR shared between restimulated and naïve T cells indicate self-reactivity, also lacks underpinning.*

**Response: We disagree. We do not equate a relatively larger abundance of eluted peptides from the WT B cells with absolute larger diversities of detected peptides; our mass spectrometry data cannot account for every single eluted peptide, because as a general rule, peptides that show up at one or two copies are not considered as reliable, hence are not counted towards the diversity of identified peptide pools. Nonetheless, we removed the 4-months culture data from the current version of the manuscript.**

- *The legend to Fig. 2 should be clarified.*

**Response: We have clarified the legend Fig 2=current Fig 3. Thank you for the suggestion.**

•	*The presentation of data concerning responses to the collagen peptide is highly confusing. As cited by the authors, this peptide is DM-sensitive, i.e. its presentation is inhibited by DM. Since DO acts as inhibitor of DM according to solid in vitro data, its absence will enhance DM editing of the collagen peptide and thereby reduce its presentation. This is discussed in the context of thymic selection, considering the expression of DO limited to TECs and B cells. Surprisingly the authors then contend that the peptide will be presented in higher numbers in DO ko mice, without justifying this notion. The paper the reports slightly though not statistically significant higher numbers of T cell precursors recognizing the collagen peptide, as well as increased numbers after immunization and higher contacts of specific T cells with damaged collagen in vivo. To remain consistent with the (surprising) notion that the collagen peptide is presented in higher numbers in the ko mice, they then propose that higher presentation by peripheral APCs is responsible for greater T cell stimulation. As this is contrary to the lack of expression of DO in peripheral macrophages and dendritic cells, this interpretation would at least require a demonstration that DO-expressing cells are key to stimulating collagen-reactive T cells.*

**Response: With all due respect to the reviewer, the point of this study is based upon previous work which suggested that DO does not simply work as an inhibitor of DM. Our hypothesis, therefore, was that DO is NOT working as an inhibitor of DM. While the body of in vitro data has offered support for the inhibitor model, the most recent paper by Denzin *et al.* [3] showed that not all of the DO constructs, identified from human subjects, conform to the strict DO inhibiting DM dogma. This finding highlights the shades of gray which exist in understanding the *in vivo* function of DO.**

**It was not, therefore, “surprising” to us that B cells in DO-KO mice could present the CII peptide at higher levels. It is also important to note that DO is not restricted exclusively to TECs and B cells. Fallas *et al*. [4] found that in mice DO is expressed in all CD11c+ DCs. Furthermore, studies using human tonsil [5] found DO expressed in CD8α DCs.**

*•	Like the collagen model, the EAE model provides evidence suggesting that H2O is indeed modulating autoreactive responses, partly using interesting methods for monitoring of autoreactive T cells in vivo. However also in this model the interpretation is largely speculative and little convincing or even contrary to known facts about H2O expression and function. The authors expect the MOG peptide to be DM-resistant, however actually measuring its editing by DM would render data interpretation much more convincing. Globally the findings in the two models of autoimmune pathologies are consistent with the notion suggested by strong literature data (though still requiring experimental validation) that H2O limits editing of low affinity self-peptides by H2M in the thymus, thereby enhancing negative CD4+ T cell selection. The authors seem to prefer an alternative model in which H2O modulates antigen presentation in the periphery, however without providing any convincing evidence for such a model.*

**Response:** **Per reviewer’s comment, we tried to address that MOG/I-A^b^ epitope is DM-insensitive experimentally. Purifying DM from mice would have been a long toll because per our experience working with HLA-DM, antibody affinity purification that requires harsh elution conditions causes loss of function in DM. Soluble H-2M has been originally produced by Dave Fremont, who informed us that he no longer had any sH-2M available. In addition, there is very few publications that have used murine DM successfully; we know of only one, from Unanue and Fremont back in early 2000s [2]. A second issue was getting some soluble I-A^b^, which we did from the NIAID Tetramer Facility and tried to perform DM-sensitivity experiments using a DM mimic peptide, AE206 Helper peptide [1]. Sadly, after much time spent on experiments in vain, we found that I-A^b^ from the Tetramer Facility has never been successfully peptide exchanged!**

**Reviewer #2:**

- *The report by Welsh et al analyses the role of HLA-DO or H2-DO in T cells selection and autoimmunity. HLA-DO or H2-DO is a non-classical MHC class II (MHCII) molecule that binds HLA-DM or H2-DM and blocks its catalytic activation and/or cooperates with DM for effective epitope selection. DM is also a non-classical MHCII molecule, which stabilizes and binds MHCII molecules to promote high affinity peptides loading for MHCII antigen presentation.  In contrast to DM, DO expression is restricted to B cells and medullary epithelial cells (mTEC), antigen-presenting cells, which remove auto reactive T cells in the thymus. Thus, the absence of DO should result in a different outcome of negative selection and also impact the presentation of self-peptides by B cells and mTECs.  This work is well written, interesting and new but relies on the use of B cells instead of mTECs.*

**Response:** **We thank this reviewer for the kind words.**

- *Page 10 the authors write: “Use of B cells instead of thymic medulla was justified for the expected similarity in antigen processing due to the expression of DO” but no reference is mentioned. Is antigen processing and presentation (antigen uptake, MHCII trafficking, proteases activity, DM, DO expression, Ii cleavage) really similar between these two APCs type? Could the authors provide some evidence for this, as it would greatly strengthen the paper?*

**Response: Thank you for the suggestion. We apologize for not including references to the known similarities between antigen processing machinery in mTEC and B cells. In the revised version of the paper, multiple references are listed that elegantly demonstrates the similarities.**

- *-Would peptides eluted from DO-/-mTECs have also like DO-/- B cells worse affinity than that CLIP for IAb?*

**Response: Yes, per similarities in the antigen processing machinery present in mTEC and B cells, we expect them to be similar. Please note that we have changed the comparisons of eluted peptides from better or worse than CLIP to absolute Affinity Scores.**

- *-What is the number of CD4+ and Treg cells in mice deficient for DO?*

**Response: In the revised manuscript, we have added a new figure (FIG 2) showing the total numbers of splenocytes, CD4s and CD8 T cells in naïve DO-WT and DO-KO mice. In addition, in Fig. 2 we have provided data enumerating Tregs in either strain, showing the presence of a larger number of Tregs in the DO-KO mice, although DO-KO Tregs did not exhibit enhanced suppressive abilities in a conventional suppression assay.**

- *In the CIA model, can the authors measure the CII-specific antibody response and score the disease?*

**Response: We tried a pilot experiment in some of our old immunized CIA diseased mice and looked for anti CII Abs (IgG2b), but did not see any differences in titers. In light of having the in vivo NIRF imaging, which specifically targets the damaged collagen and reveals the CD4 infiltration to the site of damage, we did not further pursue scoring the disease based on Ab titers.**

- *-Will it be possible to test CII antigen presentation in DR1+DO-/-mice?*

**Response: Yes, indeed, current Figure 4B shows in vivo expansion of CII specific CD4 T cells to immunization to collagen II protein as measured by tetramer positive CD4 T cell staining. We believe that this experiment shows CII antigen presentation by DR1 molecules in vivo.**

***Reviewer #3***

*The study by Welsh et al addresses the role of the non-classical MHC class II molecule H2-O in susceptibility to the development of autoimmune disease. Using two different mouse models of autoimmunity, the authors show that the absence of H2-O increases disease susceptibility in two autoimmune disease models. The effects of H2-0 are attributed to, in a peptide-specific manner, differences in epitope presentation densities or in T cell selection. There are important nuances of H2-O function brought out by the study- peptide-specific effects on epitope presentation density or T cell selection. Overall, the studies shed light on the in vivo functions of H2-O and have implications for better understanding of known HLA-DO associations with human disease.*

*While the results on the mouse model of disease are quite clear-cut, the interpretations and language is confusing to the reader in some sections of the paper, which should be addressed as outlined below. There are some other experimental points that should also be addressed. Overall, an important study.*

**Response: Thank you very much for the kind words.**

**Specific points**

*Figure 1A: Did the individual replicates conform to the overall trend shown? It would be useful to show the individual data as well as the combined data.*

**Response: Unfortunately, we did not analyze each experiment individually.**

- *Figure 1B: predicted affinities are of limited value in the absence of experimental data. Figure 1B should be deleted and in the absence of direct experimental data, some statements of the discussion are not justified (for example, “Peptides eluted from DO-KO B cells, however, conformed to an overall poorer binding affinity than CLIP for I-Ab. This finding suggests that in the absence of DO, DM is not narrowing the peptide repertoire to only those with high binding affinities”). The focus should be on the overall similarities of the results of Figure 1A with data from reference 43.*

**Response: In ref 43, authors used a human lymphoma B cell line expressing DR1 for their study, whereas, our peptide elution utilized primary murine naïve B cells expressing I-A^b^, hence, the nature of eluted peptides are different. Moreover, we are not able to test a selected number of eluted peptides experimentally (as authors in Ref 43 did) for DM-sensitivity, because of problems obtaining murine DM and functional I-A^b^ (see comments to Reviewer 1). In the absence of Data from I-A^b^ the best we could do is to rely on the IEDB algorithms. Authors in Ref 43 did not do any DM-sensitivity experiments with I-A^b^ -eluted peptides, they only stated similarities in the observed trend with human lymphoma B cell line eluted peptides. In ongoing work, we plan to used our DR1+DO-KO mice as the source for peptide elution studies.**

- *Results of Figure 2B, left panel are very interesting, particularly compared to Figure 2B, right panel- the averaged data from multiple experiments should also be shown.*

**Response: Point well taken. We have now added data from three independent experiments shown in the new Fig3 B.**

- *Figures 4A and 4B-representative data from how many experiments?*

**Response: NIRF Imaging Figures (new Fig 5 A-B=old Fig 4 A-B) have been revised and clarified to represent multiple (2 times CMP and CII tetramer injections, and -3 times for CMP and CD4 injection).**

- *Figure 6D has a legend but not an associated Figure.*

**Response: It is fixed now. Apologies for the error.**

- *“less than optimal editing by DM in the absence of DO” model (page 20 lines 315-316) should increase peptide diversity not reduce diversity. This sentence should be clarified or removed.*

**Response: it is corrected**

- *Page 20, lines 316-317:  Not clear how a lower diversity of eluted peptides from DO-KO is expected to lead to a three-fold higher level of DO-WT CD4 T cell proliferation. This sentence should also be clarified or deleted. Figure 2A types of experiments could be dominated by a few highly proliferative clones.*

**Response: It is corrected; our apologies for the error.**

**REFERENCES CITED**

1.	Chou CL, Mirshahidi S, Su KW, Kim A, Narayan K, Khoruzhenko S, et al. Short peptide sequences mimic HLA-DM functions. Mol Immunol. 2008;45(7):1935-43. Epub 2007/12/07. doi: S0161-5890(07)00835-8 [pii]

10.1016/j.molimm.2007.10.033. PubMed PMID: 18054387.

2.	Pu Z, Lovitch SB, Bikoff EK, Unanue ER. T cells distinguish MHC-peptide complexes formed in separate vesicles and edited by H2-DM. Immunity. 2004;20(4):467-76. Epub 2004/04/16. PubMed PMID: 15084275.

3.	Denzin LK, Khan AA, Virdis F, Wilks J, Kane M, Beilinson HA, et al. Neutralizing Antibody Responses to Viral Infections Are Linked to the Non-classical MHC Class II Gene H2-Ob. Immunity. 2017;47(2):310-22 e7. Epub 2017/08/17. doi: 10.1016/j.immuni.2017.07.013. PubMed PMID: 28813660; PubMed Central PMCID: PMCPMC5568092.

4.	Fallas JL, Yi W, Draghi NA, O'Rourke HM, Denzin LK. Expression patterns of H2-O in mouse B cells and dendritic cells correlate with cell function. J Immunol. 2007;178(3):1488-97. Epub 2007/01/24. doi: 178/3/1488 [pii]. PubMed PMID: 17237397.

5.	Hornell TM, Burster T, Jahnsen FL, Pashine A, Ochoa MT, Harding JJ, et al. Human dendritic cell expression of HLA-DO is subset specific and regulated by maturation. J Immunol. 2006;176(6):3536-47. PubMed PMID: 16517722.
